# Traumatic cardiac rupture with cerebral herniation: A case report

**DOI:** 10.1097/MD.0000000000047259

**Published:** 2026-01-16

**Authors:** Yi Zhu, Xinhe Huang, Zheng Zhu, Baisheng Xie, Jun Wang

**Affiliations:** a Department of Cardiothoracic Surgery, Hangzhou TCM Hospital Affiliated to Zhejiang Chinese Medical University, Hangzhou, China; b Department of Obstetrics and Gynecology, The Second Affiliated Hospital of Zhejiang Chinese Medical University, Hangzhou, China.

**Keywords:** cardiac rupture, case report, cerebral herniation, damage-control surgery, multidisciplinary collaboration, multiple injuries

## Abstract

**Rationale:**

Traumatic cardiac rupture with concurrent cerebral herniation represents a critical emergency characterized by an extremely high prehospital mortality rate. Early diagnosis and multidisciplinary intervention are essential for survival. As this is a single-case report, the observations should be interpreted with caution.

**Patient concerns:**

A 45-year-old woman presented in a coma with hemorrhagic shock following a traffic accident. Imaging revealed a massive left hemothorax, multiple rib fractures, and a right subdural hematoma with a 10 mm midline shift.

**Diagnosis:**

Emergency surgery confirmed the diagnoses of cardiac rupture and cerebral herniation.

**Interventions:**

Damage-control surgery was initiated within 2 hours and included emergency thoracotomy for cardiac repair and decompressive craniectomy.

**Outcome:**

The patient was discharged on postoperative day 54. During follow-up, she exhibited no delayed cardiac complications on echocardiography.

**Lessons:**

This case is notable for the rare concurrence of traumatic cardiac rupture and evolving cerebral herniation, necessitating staged, back-to-back thoracotomy and decompressive craniectomy. Early recognition, multidisciplinary coordination, and adherence to damage-control sequencing were essential for survival.

## 1. Introduction

Traumatic cardiac rupture (TCR) is a rare but often fatal consequence of blunt or penetrating thoracic trauma, with 50% to 90% of patients dying before reaching the hospital.^[1,2]^ Concurrent cerebral herniation increases therapeutic complexity because hemodynamic resuscitation may exacerbate neurological injury. Few reports have described this dual pathology, leaving management protocols undefined. We report a case of polytrauma involving coexisting cardiac rupture and cerebral herniation. Contemporary polytrauma care emphasizes damage-control (DC) resuscitation, rapid identification of life-threatening hemorrhage, and early neuroprotection, in accordance with widely used guidelines.^[3,4]^ These principles guided the prioritization of thoracic and neurosurgical interventions in this unstable patient. Despite advances in trauma care, reports specifically addressing concurrent TCR and cerebral herniation remain scarce, and no standardized management pathways have been established. This case underscores the need for rapid multidisciplinary coordination at the interface between cardiothoracic stabilization and neurosurgical priorities.

## 2. Case presentation

A 45-year-old woman presented to the emergency department after a high-impact motor vehicle collision. On arrival, the patient was in critical condition. Her vital signs were as follows: Glasgow Coma Scale score, 3; blood pressure, 93/85 mm Hg; heart rate, 123 beats/min; respiratory rate, 28 breaths/min; and peripheral oxygen saturation, 91%. Prehospital emergency physicians performed endotracheal intubation because of coma and paradoxical breathing.

**Figure 1.**
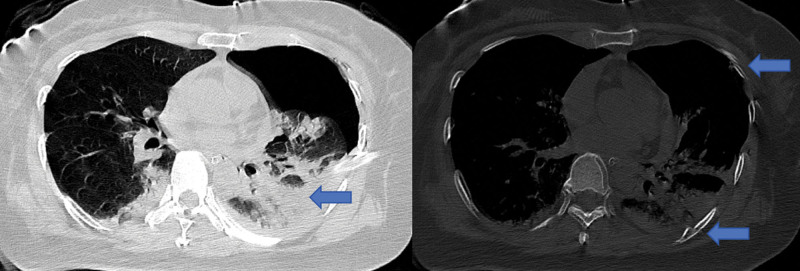
Thoracic CT findings. Contrast-enhanced thoracic computed tomography (CT) images showing left-sided hydropericardium (solid arrow), haemopneumothorax (asterisk), pulmonary contusion (arrowheads), and multiple rib fractures (circles). These findings indicate major blunt thoracic trauma with active intrathoracic bleeding. CT = computed tomography.

**Figure 2.**
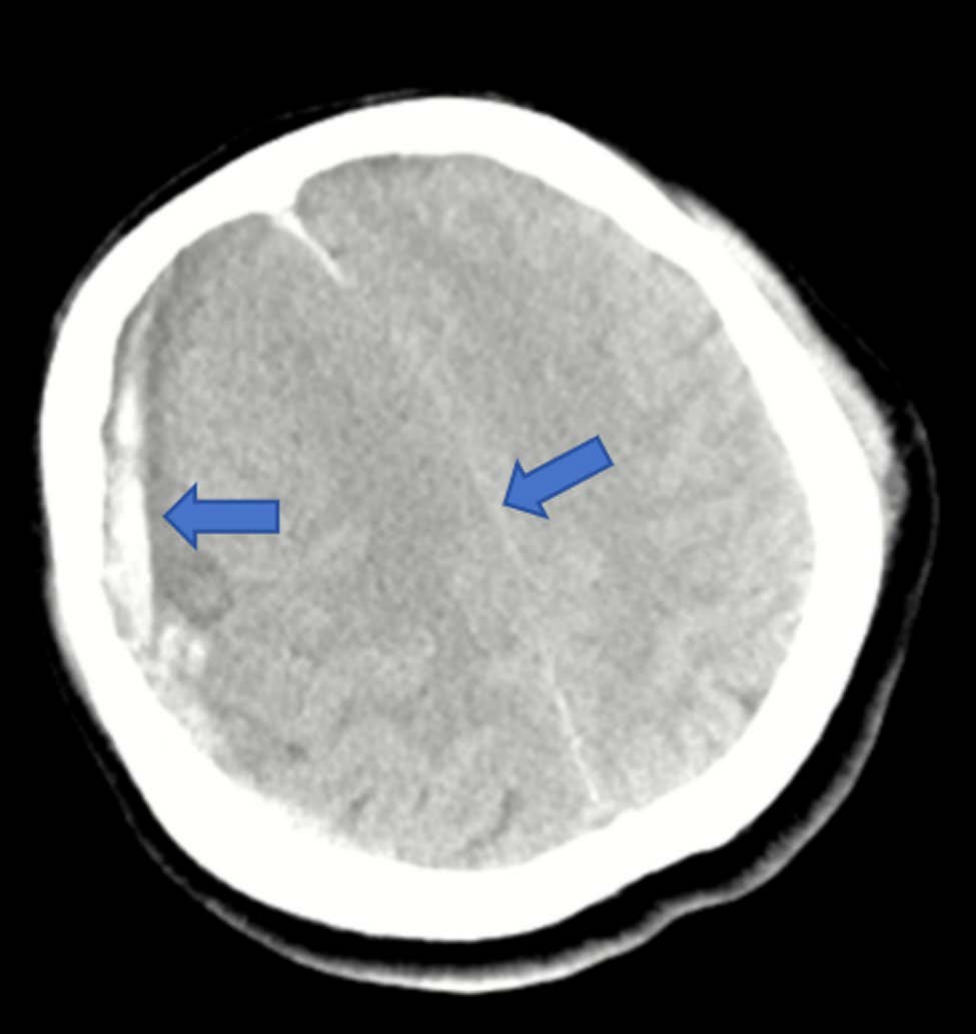
Cranial CT findings. Non-contrast cranial CT demonstrating a right subdural hematoma (arrow) producing a 10-mm midline shift. Bilateral pulmonary contusions are also visible on the same scan series. CT = computed tomography.

The focused assessment with sonography for trauma (FAST) was positive in the thoracic window, revealing a large left haemothorax (9.7 × 6.9 cm), but negative in the cardiac window. A whole-body computed tomography (WBCT) scan was subsequently performed. The WBCT revealed hydropericardium, hemopneumothorax, lung contusion, and multiple left-sided rib fractures (Fig. 1), as well as a right-sided subdural hematoma with a 10 mm midline shift and bilateral pulmonary contusions (Fig. 2). Laboratory tests showed severe metabolic acidosis (pH 7.26, lactate 11.5 mmol/L) and a markedly reduced hemoglobin concentration of 74 g/L. Advanced airway management, intraosseous access, vasopressor support, and activation of a massive transfusion protocol (initiating 4 units of packed red blood cells) were instituted.

**Figure 3.**
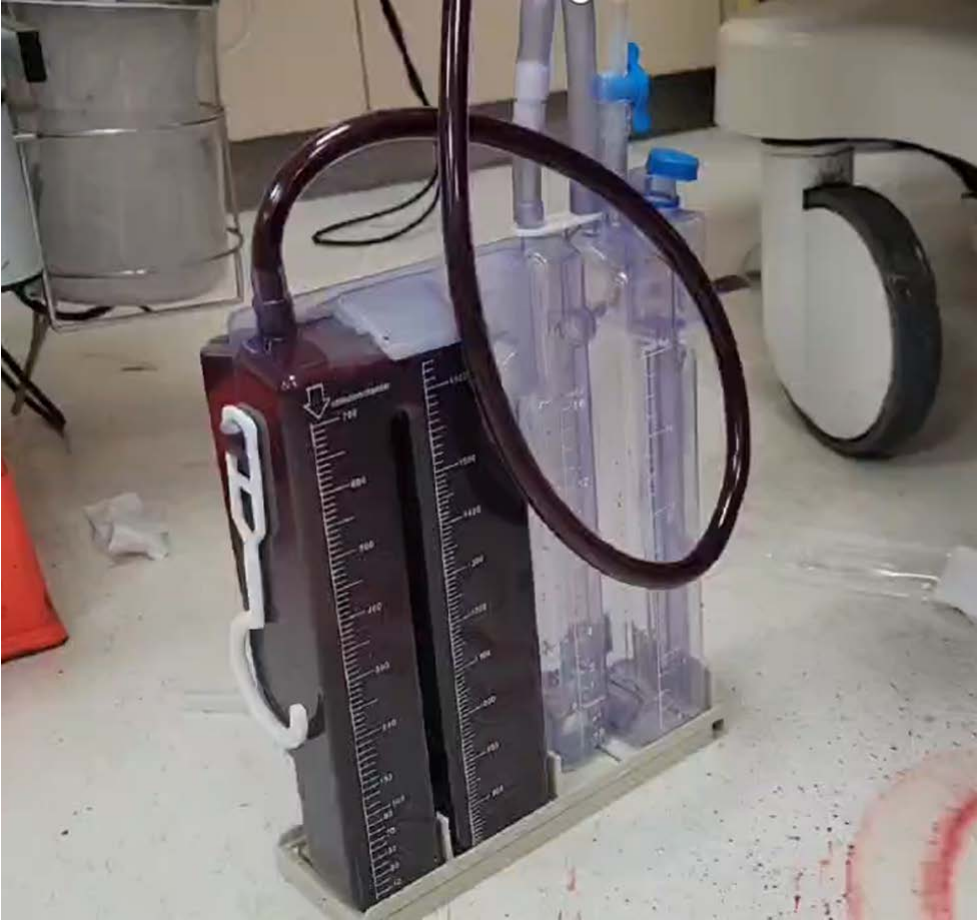
Chest-tube drainage. A large volume of blood-stained pleural effusion was drained through a left thoracic tube (arrow). Prompt clamping and preparation for autologous blood reinfusion were undertaken to facilitate immediate autotransfusion.

A chest tube was inserted, draining a large volume of blood-stained pleural fluid (Fig. 3). The chest tube was promptly clamped, and an autologous blood reinfusion system was prepared for immediate autotransfusion. Concurrently, the neurosurgeon placed an external ventricular drain (EVD) at the bedside under ultrasound guidance, which revealed an intracranial pressure (ICP) of 15 mm Hg. Despite aggressive resuscitation, hemodynamic instability persisted, characterized by refractory hypotension and worsening metabolic acidosis. This clinical trajectory necessitated an immediate transition to damage-control surgery (DCS).

**Figure 4.**
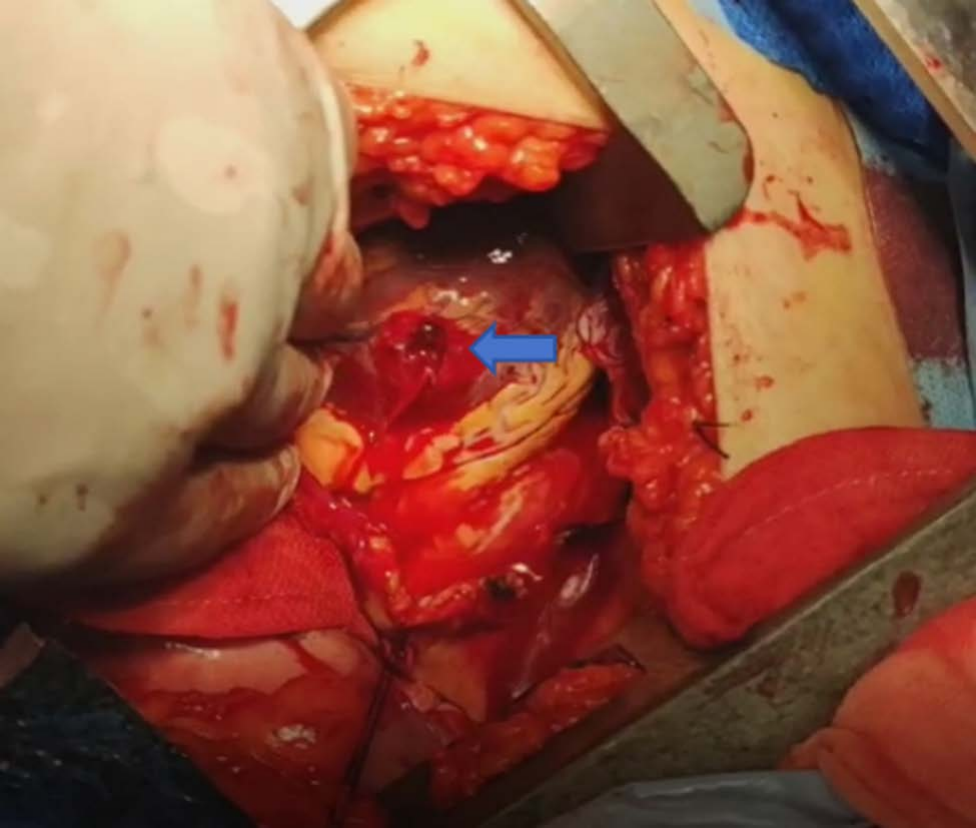
Intraoperative view of cardiac rupture. Intraoperative photograph showing active blood ejection (arrow) from a 1 × 0.5 cm full-thickness laceration at the anterior wall of the left ventricle. LV = left ventricle.

**Figure 5.**
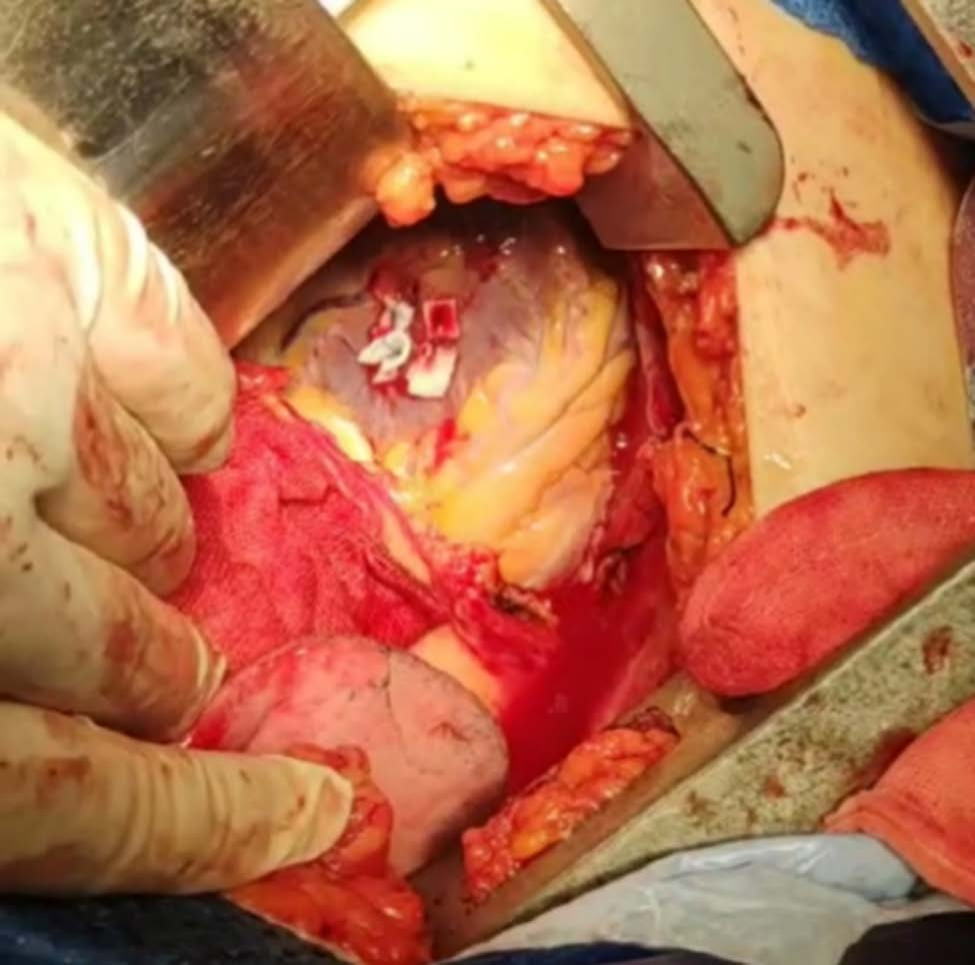
Surgical repair of cardiac rupture. Operative field after repair of the left-ventricular rupture with 4-0 polypropylene sutures and nylon pledgets using an interrupted horizontal-mattress technique. Hemostasis was achieved and pericardial integrity restored.

**Figure 6.**
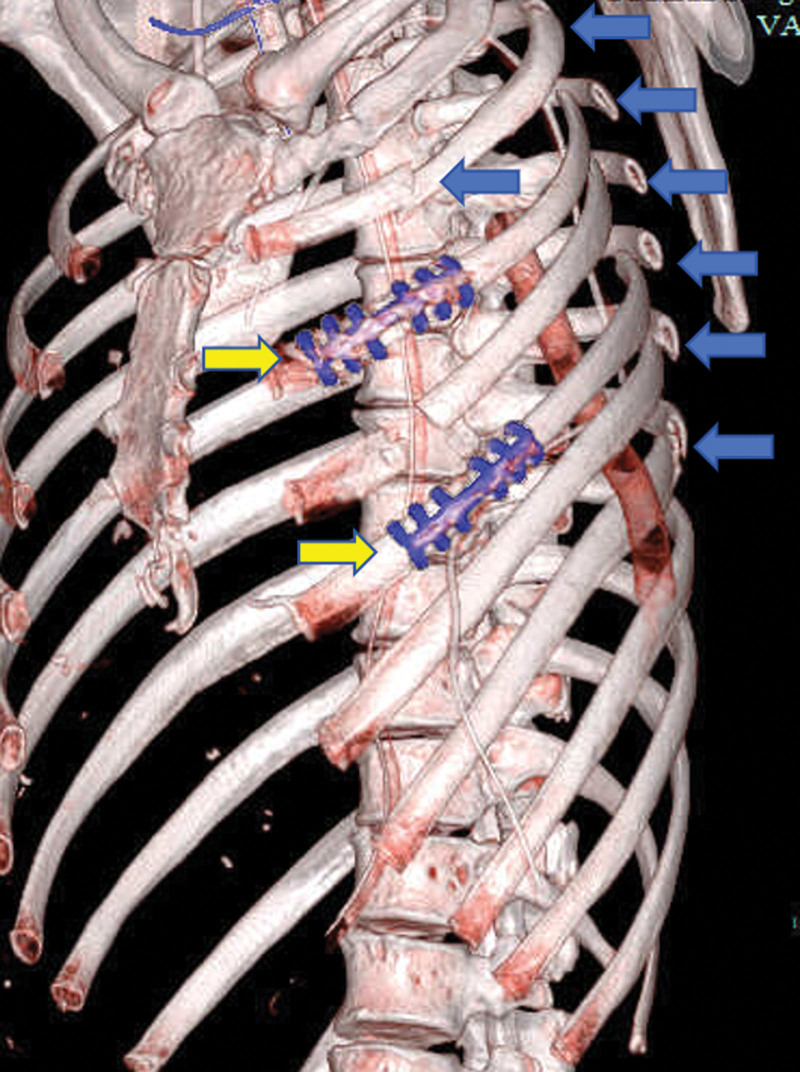
Rib-fracture stabilization. Multiple displaced rib fractures (blue arrows) were identified intraoperatively. To prevent secondary injury during positional change, temporary fixation was performed at the cranial and caudal edges of the incision (yellow arrows) before neurosurgical repositioning.

**Figure 7.**
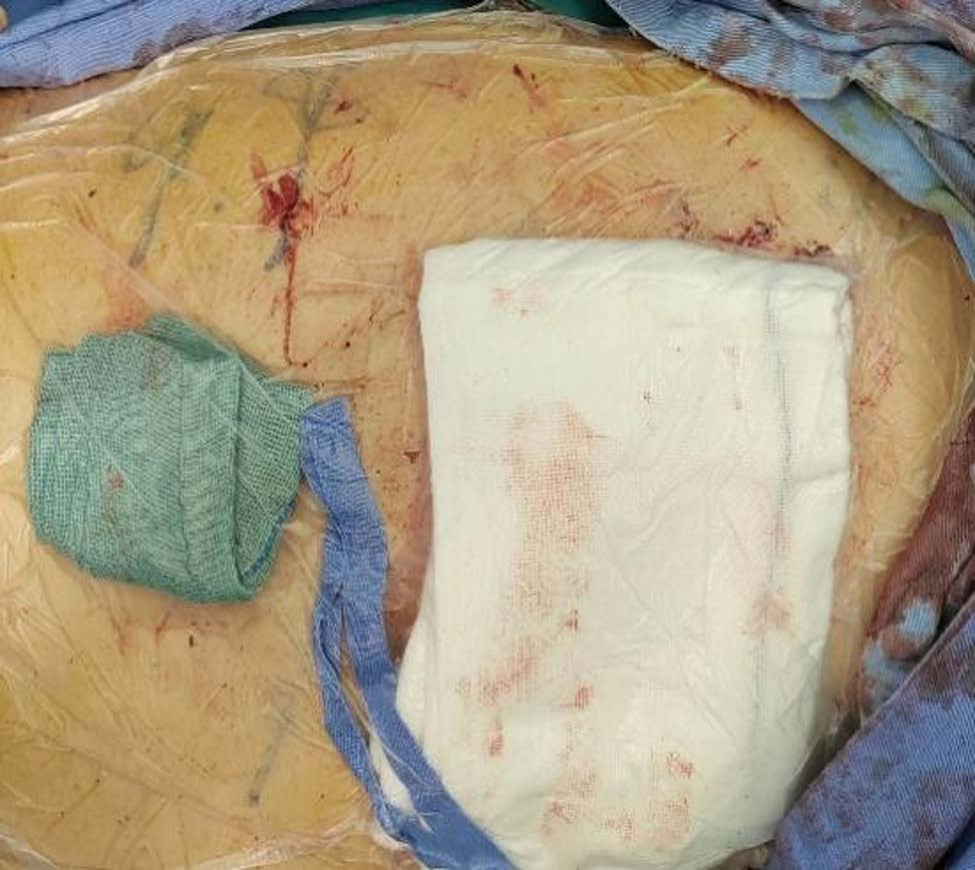
Temporary chest closure. Because of progressive intracranial hypertension and cerebral herniation, the thoracic cavity was temporarily sealed with sterile occlusive dressings to maintain asepsis during the transition to neurosurgical intervention.

**Figure 8.**
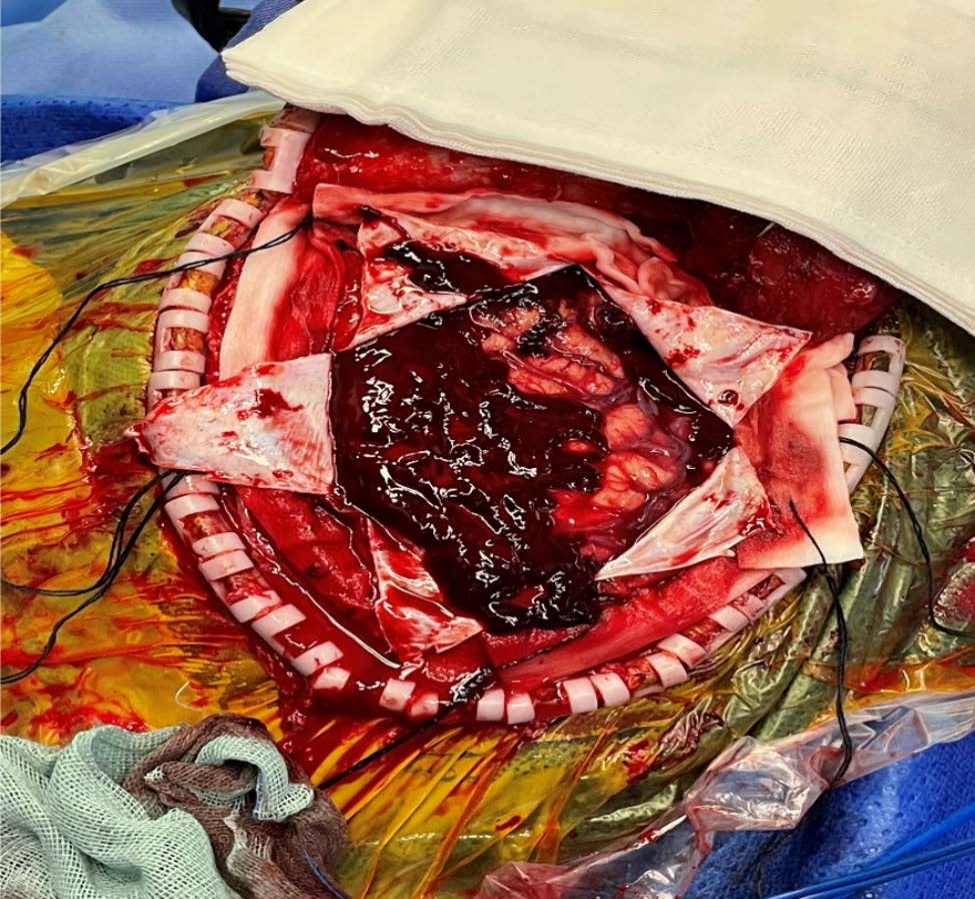
Decompressive craniectomy. Intra-operative image showing evacuation of a 20-mL right subdural hematoma (arrow) during decompressive craniectomy. SDH = subdural hematoma.

After induction of general anesthesia, the patient was positioned in the right lateral decubitus position. A left-sided exploratory thoracotomy was performed through the fourth intercostal space along the midaxillary line. Intraoperative exploration revealed a massive haemothorax and a 2-cm longitudinal pericardial laceration. Upon pericardiotomy, a large amount of organized clot was evacuated. Two active bleeding sources were identified: a 1.0 × 0.5 cm full-thickness myocardial rupture in the anterior wall of the left ventricle (Fig. 4) and a 0.5 cm linear tear in the anterior wall of the left superior pulmonary vein. Both defects were repaired with 4-0 polypropylene sutures buttressed with nylon pledgets, using an interrupted horizontal mattress technique (Fig. 5). Systematic exploration revealed no injury to the pulmonary parenchyma, diaphragm, or major vasculature. After completing the cardiac repair, we prepared to perform internal fixation of the rib fractures. However, acute intracranial hypertension developed, characterized by an abrupt rise in ICP to 45 mm Hg and hemodynamic collapse (blood pressure 70/40 mm Hg), raising concern for progressive cerebral herniation. To prevent secondary injury from unstable rib fractures, we temporarily stabilized the fractured ends at the cranial and caudal margins of the incision and deferred definitive wound closure (Fig. 6). The thoracic cavity was temporarily sealed with sterile occlusive dressings to maintain asepsis during this critical transition between surgical teams (Fig. 7). The patient was then urgently repositioned from the right to the left lateral decubitus position to facilitate emergent neurosurgical intervention. The neurosurgical team performed a decompressive craniectomy with evacuation of a right-sided intracranial hematoma. Intraoperatively, a 20 mL right subdural hematoma was confirmed (Fig. 8).

**Figure 9.**
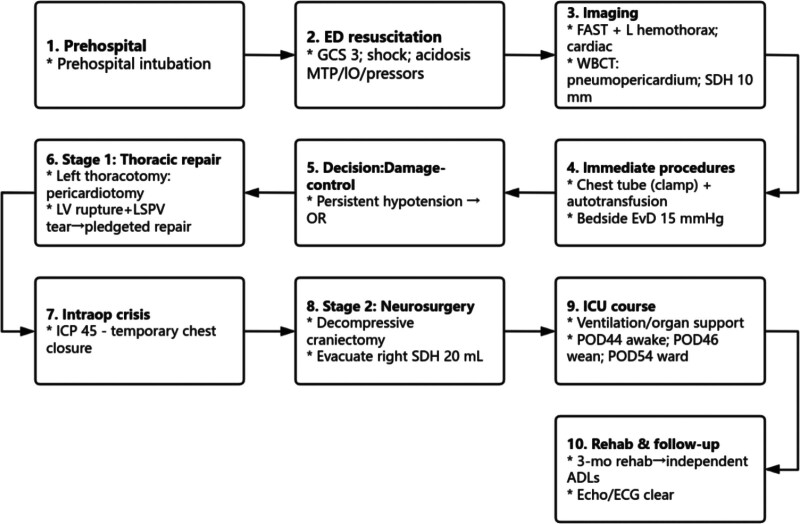
Flowchart of clinical management. Algorithm summarizing the sequence of multidisciplinary interventions from prehospital resuscitation to postoperative rehabilitation. The chart includes: (1) initial assessment (FAST screening → WBCT confirmation of thoracic and cranial injuries); (2) damage-control resuscitation (massive transfusion, airway and hemodynamic support); (3) emergency thoracotomy (pericardial exploration → cardiac repair); (4) detection of acute intracranial hypertension → bedside EVD placement; (5) transition to neurosurgery → decompressive craniectomy; (6) postoperative intensive-care support → ventilator weaning → rehabilitation and follow-up. EVD = external ventricular drain; FAST = focused assessment with sonography for trauma; ICP = intracranial pressure; VA-ECMO = veno-arterial extracorporeal membrane oxygenation; WBCT = whole-body computed tomography.

Postoperatively, the patient received mechanical ventilation, antimicrobial therapy, comprehensive organ support, and maintenance of hemodynamic and metabolic homeostasis. The patient regained consciousness on postoperative day (POD) 44, followed by successful ventilator weaning on POD 46. On POD 53, the patient was able to follow basic commands and was transferred to the general ward the following day (POD 54). After 3 months of functional rehabilitation at an external facility, the patient achieved independence in activities of daily living. At follow-up, serial echocardiography and electrocardiography revealed no delayed complications, such as ventricular septal defect or ventricular aneurysm. At present, the patient has no significant organ dysfunction except for a minor left-sided chest-wall deformity. Thoracotomy was initially prioritized to control exsanguination in refractory shock while early EVD placement was initiated for ICP monitoring. With neurological deterioration and concern for rising ICP, decompressive craniectomy was performed in a staged, DC sequence. The complete timeline of the clinical course is presented in Table 1 and Figure 9.

**Table 1:** Timeline of clinical events.

Phase	Time/day	Action/intervention	Key findings/details	Rationale/notes
Prehospital	Scene → ED	Endotracheal intubation	Coma with paradoxical breathing	Airway protection prior to transport
ED arrival	Hour 0	Primary survey; resuscitation; MTP initiated	GCS 3; BP 93/85 mm Hg; HR 123/min; RR 28/min; SpO_2_ 91%; Hgb 74 g/L; pH 7.262; lactate 11.5 mmol/L	Shock with severe metabolic acidosis → early blood products (4U PRBC), IO access, vasopressors
Initial imaging	Hour 0–1	FAST + chest windows; cardiac window negative	Massive left hemothorax (9.7 × 6.9 cm); no pericardial effusion on initial cardiac view	Suggests thoracic bleeding; maintain suspicion for cardiac injury
WBCT	Hour 1	Whole-body CT	Pneumopericardium; hemopneumothorax; left rib fractures; lung contusion; subdural hematoma with 10 mm midline shift; bilateral pulmonary contusions	Defines thoracic + neuro injuries to guide simultaneous plans
ED procedures	Hour 1–2	Left chest tube; autotransfusion setup; bedside EVD	Large-volume bloody drainage; EVD ICP 15 mm Hg	Drain/recycle blood; continuous ICP monitoring while resuscitation proceeds
Deterioration	Early hours	Decision for damage-control surgery	Persistent hypotension and worsening acidosis	Proceed to OR for hemorrhage control
Thoracotomy	OR (Stage 1)	Left anterolateral thoracotomy; pericardiotomy; pledgeted repairs	2 cm pericardial laceration; LV anterior wall rupture (1.0 × 0.5 cm); left superior pulmonary vein tear (0.5 cm)	Hemorrhage control; definitive repair of cardiac injuries
Intraop ICP surge	During Stage 1	Temporary chest closure; defer definitive chest/rib fixation	ICP spike to 45 mm Hg with hemodynamic collapse	Suspected cerebral herniation → staged approach
Neurosurgery	OR (Stage 2)	Decompressive craniectomy + evacuation of right SDH	20 mL right subdural hematoma evacuated	Control intracranial hypertension and secondary brain injury
ICU course	POD 0–54	Ventilation, anti-infective therapy, organ support	Consciousness regained POD 44; ventilator weaning POD 46; obeys basic commands POD 53; transfer to ward POD 54	Gradual neurologic recovery with organ support
Rehabilitation	3 months	Structured multidisciplinary rehab	Independent activities of daily living	Functional recovery
Follow-up	Outpatient	Serial echo and ECG	No VSD, aneurysm, or significant arrhythmia; minor left chest wall deformity	No delayed cardiac complications

BP = blood pressure, CT = computed tomography, ECG = electrocardiogram, ED = emergency department, EVD = external ventricular drain, FAST = focused assessment with sonography for trauma, GCS = Glasgow Coma Scale, Hgb = hemoglobin, HR = heart rate, ICP = intracranial pressure, ICU = intensive care unit, IO = intraosseous, LV = left ventricle, MTP = massive transfusion protocol, OR = operating room, pH = potential of hydrogen, POD = postoperative day, PRBC = packed red blood cells, RR = respiratory rate, SDH = subdural hematoma, SpO₂ = peripheral oxygen saturation, VSD = ventricular septal defect, WBCT = whole-body computed tomography.

## 3. Discussion

Cardiac rupture with concurrent cerebral herniation is a rare but often fatal consequence of high-energy blunt trauma, as illustrated by the present case following a severe motor vehicle collision. Cardiac rupture carries a mortality rate exceeding 80%, with most patients dying before a definitive diagnosis can be established.^[5]^ The coexistence of cerebral herniation further increases therapeutic complexity, as intracranial hypertension exacerbates systemic instability and complicates the prioritization of surgical interventions. In addition, hypoxemia secondary to chest trauma represents an additional insult that may aggravate traumatic brain injury (TBI).^[6]^

Although isolated cardiac injuries are well documented, reports of concurrent cardiac rupture and cerebral herniation remain scarce, and no standardized management protocols have been established for such complex polytrauma.

Diagnosing cardiac trauma is clinically challenging because pathognomonic features are often absent, and no definitive gold standard exists. Cardiac injury typically manifests in 3 pathophysiological forms: pericardial tamponade, acute blood loss, and impaired myocardial function.^[7]^ Although life-threatening, the compressive effect of pericardial tamponade may provide a short-term survival advantage in acute cardiac trauma by temporarily stabilizing myocardial tears through extrinsic hemostatic pressure.^[8]^ Previous studies have suggested that FAST can facilitate diagnostic evaluation in suspected cardiac injury.^[9]^ In our case, cardiac tamponade was absent, and the initial ultrasound examination yielded negative findings. Nevertheless, serial ultrasonographic surveillance remains essential even after an initially negative assessment, as delayed manifestations of cardiac injury may develop over time. Intraoperative exploration corroborated this finding, revealing a pericardial rupture that allowed direct hemorrhage into the thoracic cavity, without the classic manifestations of Beck triad. Therefore, patients with trauma involving the precordial region should always be evaluated for potential cardiac rupture. To address diagnostic uncertainty, we emphasize the complementary roles of serial focused cardiac ultrasound and intraoperative transoesophageal echocardiography. Because pericardial tears can decompress tamponade into the pleural space and result in false-negative pericardial views, focused echocardiography should be repeated after initial resuscitation and again immediately before or during thoracotomy.^[10,11]^

When definitive operative management is anticipated, transoesophageal echocardiography provides a rapid bedside assessment of ventricular function, wall defects, and valvular injury without interrupting resuscitation. CT angiography is reserved for use in hybrid emergency suites or after transient stabilization, whereas routine transfer to CT is avoided in persistently unstable patients.^[9,12]^ In general, for severely traumatized patients, control of torso hemorrhage takes priority over the management of brain injury.^[13]^ However, in this case, CT findings necessitated bedside placement of an EVD under ultrasound guidance, which revealed an ICP of 15 mm Hg. Because CT demonstrated a large subdural hematoma with midline shift and the patient required urgent thoracic hemorrhage control, a bedside EVD was placed to provide therapeutic cerebrospinal fluid diversion and continuous ICP monitoring without the need for transport. This approach enabled goal-directed management of ICP and cerebral perfusion pressure during resuscitation. Delaying cerebrospinal fluid diversion could have resulted in intracranial hypertension and perioperative decompensation; therefore, bedside EVD placement represented the safest bridge to definitive neurosurgical decompression.^[14,15]^ WBCT plays a critical role in the initial assessment of trauma patients, and multiple clinical studies have demonstrated an association between its use and improved survival.^[16,17]^

Reports of blunt cardiac rupture coexisting with severe TBI or cerebral herniation are rare but consistently highlight 2 recurring themes: diagnostic pitfalls (particularly false-negative pericardial FAST when a concomitant pericardial tear decompresses tamponade into the pleural space^[11]^); and determinants of survival (namely, time to definitive exposure [anterolateral thoracotomy or sternotomy] and prompt pledgeted repair).^[18]^ Our case mirrors these patterns, featuring an initially negative ultrasound in the setting of massive haemothorax, followed by a decisive left anterolateral thoracotomy and staged, DC sequencing. These literature-based insights contextualize our management decisions and help explain the favorable outcome achieved in this hemodynamically unstable patient.

While several series and reviews emphasize the value of rapid exposure, pledgeted repair, and abbreviated DC tactics in hemodynamically unstable patients, other reports highlight substantial early mortality and major complications with thoracic DC strategies.^[19–24]^ For instance, DC thoracotomy and temporary chest closure can be life-saving in marginal physiology yet have been associated with significant complications and high early attrition in some cohorts.^[22,23]^ Similarly, although many protocols favor primary sternotomy in stable settings, an anterolateral thoracotomy may prioritize speed and immediate access to left-sided injuries when profound shock precludes transfer or more extensive exposure; however, this lateral approach risks limited visualization of concomitant lesions and may necessitate reexploration.^[24,25]^ Beyond the index operation, extracorporeal support illustrates further trade-offs: veno-arterial extracorporeal membrane oxygenation can bridge refractory cardiogenic shock after initial repair, and veno-venous extracorporeal membrane oxygenation may support severe hypoxemia from lung contusion or acute respiratory distress syndrome once bleeding is controlled, but outcomes are mixed, anticoagulation often must be minimized or delayed, and resource intensity is considerable.^[26,27]^ Notably, while isolated reports have described either TCR or cerebral herniation, concurrent survival from both injuries remains exceedingly rare. For example, Chen et al^[18]^ reported a case of blunt cardiac rupture presenting as massive hemothorax, but the patient died shortly after arrival due to delayed recognition and missed pericardial injury. Similarly, in the cohort described by Khurshid et al,^[23]^ more than half of patients requiring thoracic DCS (many with concomitant traumatic brain injuries) died within 24 hours or during hospitalization from complications such as coagulopathy, sepsis, or multiorgan failure. Compared with these outcomes, the present case demonstrated a markedly better prognosis, likely attributable to timely EVD placement, rapid left anterolateral thoracotomy with pledgeted repair, and immediate transition to neurosurgical decompression. This coordinated, staged approach may account for the patient’s favorable neurological and cardiac recovery despite dual life-threatening injuries. Finally, diagnostic pathways are not uniformly protective: pericardial tears may decompress tamponade into the pleural space, yielding false-negative pericardial FAST, and delaying recognition.^[9,11]^ Taken together, these data suggest that our chosen sequence (rapid left anterolateral thoracotomy with pledgeted repair, deferred closure, and staged neurosurgical decompression) should be viewed as one context-dependent option among several, rather than a universally superior pathway.

DC techniques have been employed by surgeons for more than a century. However, the concept of DCS did not take formal shape until the 1980s.^[19]^ In patients requiring operative intervention after major trauma, surgeons must decide between performing a definitive procedure or adopting a DC approach.^[20]^ In our case, the abrupt rise in ICP after cardiac repair (an unpredictable event) highlighted the dynamic interplay between systemic hemodynamics and cerebral perfusion. The favorable outcome largely depended on the implementation of a DC strategy. The initial thoracotomy prioritized hemorrhage control and cardiac repair, whereas deferring definitive closure enabled an immediate transition to craniotomy for hematoma evacuation and decompression. This staged approach minimized the “second hit” associated with prolonged surgery, thereby attenuating systemic inflammatory responses and mitigating secondary brain injury.

Thoracic packing and temporary chest closure remain controversial strategies for the management of critically ill patients with severe chest trauma.^[21]^ Our case suggests that temporary thoracic closure to restore physiological stability before definitive surgery may represent a viable strategy in patients with unstable thoracic trauma. A similar conclusion was reported by Douglas et al.^[22]^

Although Khurshid et al reported that one-third of patients undergoing thoracic DCS died within 24 hours, more than half developed major complications and subsequently died during hospitalization.^[23]^ This patient clearly benefited from the application of DCS. Our strategy (urgent left anterolateral thoracotomy, controlled pericardiotomy, and pledgeted polypropylene repair) was consistent with established trauma protocols and published case series on suspected blunt cardiac rupture.^[24]^ These sources consistently emphasize rapid exposure (via anterolateral thoracotomy or median sternotomy), avoidance of coronary compromise during repair, and abbreviated DC tactics with temporary chest closure when physiological status is marginal. They also caution against delaying definitive repair for nonessential imaging in hemodynamically unstable patients. Compared with protocols favoring primary sternotomy in stable cases, our lateral approach prioritized speed and direct access to the left ventricular and left pulmonary venous injuries in this hemodynamically unstable patient.^[25]^ For refractory cardiogenic shock or recurrent cardiac arrest after initial repair, veno-arterial extracorporeal membrane oxygenation may serve as a bridge to recovery, provided that surgical bleeding is controlled or controllable. Peripheral (femoral–femoral) cannulation is typically the fastest option; anticoagulation should be individualized in trauma patients (minimized or initially delayed when intracranial or thoracic bleeding risk is high) with close hematological and circuit monitoring. For refractory hypoxaemic respiratory failure resulting from lung contusion or acute respiratory distress syndrome, despite lung-protective ventilation, veno-venous extracorporeal membrane oxygenation represents an additional supportive option once major hemorrhage has been controlled.^[26,27]^ In this case, an early, goal-directed, multidisciplinary rehabilitation programme was implemented, comprising pulmonary rehabilitation (incentive spirometry and chest physiotherapy), early mobilization with progressive resistance training, occupational and cognitive therapy to support activities of daily living, and graded aerobic conditioning. These interventions reduced atelectasis and ICU-acquired weakness, facilitating the transition from assisted to independent ambulation. Therapy intensity was titrated according to hemodynamic stability, pain control, and neurological status, ensuring a safe discharge with a structured home rehabilitation plan.^[28,29]^

To our knowledge, few reported cases of multiple severe thoracic injuries complicated by TBI have required a combination of EVD placement and other invasive surgical interventions. The management of multiple trauma carries an inherent risk of attentional diversion, whereby prioritizing dominant injuries may result in diagnostic oversight of concomitant lesions. The favorable outcome in this patient likely reflected the effectiveness of timely, multidisciplinary collaboration. However, as a single-case report, our findings are not generalizable, remain vulnerable to selection, reporting, and survivorship biases, depend on center-specific resources, and lack comprehensive long-term patient-reported outcomes. The favorable outcome observed in this case may partly reflect survivorship bias, as patients with comparable dual injuries often die before hospital arrival or during early resuscitation. Furthermore, the management relied heavily on resource-intensive capabilities (including rapid access to whole-body CT, hybrid surgical capacity, and coordinated cardiothoracic and neurosurgical expertise) which may not be available in all institutions. Therefore, while this report illustrates a potential framework for multidisciplinary trauma management, its reproducibility and external applicability remain limited by institutional infrastructure and resource constraints. Multicenter prospective data are required to validate the proposed management approach.

## 4. Conclusion

TCR, particularly when combined with craniocerebral injury, represents a formidable clinical challenge due to its high mortality and diagnostic complexity. Few related studies have been reported based on our review of the literature. This case underscores the critical importance of early recognition, multidisciplinary collaboration, and adherence to DC principles in improving outcomes for such critically injured patients.

## Acknowledgments

We would like to credit the patient for her participation in this case study.

## Author contributions

**Conceptualization:** Yi Zhu, Zheng Zhu, Jun Wang.

**Data curation:** Yi Zhu, Xinhe Huang, Zheng Zhu, Jun Wang.

**Formal analysis:** Xinhe Huang, Baisheng Xie.

**Funding acquisition:** Baisheng Xie.
